# Silencing GMPPB Inhibits the Proliferation and Invasion of GBM via Hippo/MMP3 Pathways

**DOI:** 10.3390/ijms241914707

**Published:** 2023-09-28

**Authors:** Zi-Lu Huang, Aalaa Sanad Abdallah, Guang-Xin Shen, Milagros Suarez, Ping Feng, Yan-Jiao Yu, Ying Wang, Shuo-Han Zheng, Yu-Jun Hu, Xiang Xiao, Ya Liu, Song-Ran Liu, Zhong-Ping Chen, Xiao-Nan Li, Yun-Fei Xia

**Affiliations:** 1 State Key Laboratory of Oncology in Southern China, Department of Radiation Oncology, Sun Yat-Sen University Cancer Center, Guangzhou 510060, China or ziluhuang@luriechildrens.org (Z.-L.H.); fengping@sysucc.org.cn (P.F.); wangying2@sysucc.org.cn (Y.W.); zhengsh1@sysucc.org.cn (S.-H.Z.); huyj1@sysucc.org.cn (Y.-J.H.); xiaoxiang@sysucc.org.cn (X.X.); liuya8@sysucc.org.cn (Y.L.); 2 Program of Precision Medicine PDOX Modeling of Pediatric Tumors, Ann & Robert H. Lurie Children’s Hospital of Chicago, Chicago, IL 60611, USA; aabdallah@luriechildrens.org (A.S.A.); msuarezpalacios@luriechildrens.org (M.S.); 3 Department of Pediatrics, Northwestern University Feinberg School of Medicine, Chicago, IL 60611, USA; 4 Foshan Clinical Medical School of Guangzhou University of Chinese Medicine, Guangzhou 528031, China; sgx94120@163.com; 5 State Key Laboratory of Oncology in Southern China, Department of Neurosurgery/Neuro-Oncology, Sun Yat-Sen University Cancer Center, Guangzhou 510060, China; yuyj@sysucc.org.cn (Y.-J.Y.); chenzhp@sysucc.org.cn (Z.-P.C.); 6 State Key Laboratory of Oncology in Southern China, Department of Pathology, Sun Yat-Sen University Cancer Center, Guangzhou 510060, China; liusr@sysucc.org.cn

**Keywords:** GMPPB, glioma, hippo, invasion, proliferation, verteporfin, matrix metallopeptidase 3 (MMP3)

## Abstract

Glioblastoma multiforme (GBM) is a highly aggressive malignancy and represents the most common brain tumor in adults. To better understand its biology for new and effective therapies, we examined the role of GDP-mannose pyrophosphorylase B (GMPPB), a key unit of the GDP-mannose pyrophosphorylase (GDP-MP) that catalyzes the formation of GDP-mannose. Impaired GMPPB function will reduce the amount of GDP-mannose available for O-mannosylation. Abnormal O-mannosylation of alpha dystroglycan (α-DG) has been reported to be involved in cancer metastasis and arenavirus entry. Here, we found that GMPPB is highly expressed in a panel of GBM cell lines and clinical samples and that expression of GMPPB is positively correlated with the WHO grade of gliomas. Additionally, expression of GMPPB was negatively correlated with the prognosis of GBM patients. We demonstrate that silencing GMPPB inhibits the proliferation, migration, and invasion of GBM cells both in vitro and in vivo and that overexpression of GMPPB exhibits the opposite effects. Consequently, targeting GMPPB in GBM cells results in impaired GBM tumor growth and invasion. Finally, we identify that the Hippo/MMP3 axis is essential for GMPPB-promoted GBM aggressiveness. These findings indicate that GMPPB represents a potential novel target for GBM treatment.

## 1. Introduction

Gliomas, tumors that display histological similarities to glial cells, are the most prevalent and lethal primary tumors of the brain [1]. Glioblastoma multiforme (GBM) is the most common type of glioma, accounting for more than half of all gliomas [2]. GBM is also one of the most aggressive malignant brain and CNS histopathologies and has been categorized as a WHO grade IV glioma [2]. The median survival for patients with GBM is 14–16 months due to its significant capacity for invasion and resistance to multi-therapy, including surgical resection, radiation, and chemotherapy [3]. Despite advancements in CNS tumor therapies, the 5-year survival for GBM patients between 15 and 39 years old is approximately 25%, and for patients over 40 years old, it is less than 10% [2]. Alterations in multiple signaling pathways have been identified as part of the molecular cascades responsible for glioma tumorigenesis. In addition to well-established signaling pathways that have been characterized in the context of glioma genesis, such as PI3K/AKT/PTEN, EGFR, TP53, and RB1 [4,5,6,7], the influence of additional signaling pathways is increasingly being explored, including the Notch, Wnt, and Hippo pathways [8,9,10]. Some of these pathways, such as EGFR, have been tested as therapeutic targets [11,12]. Given the continued poor prognosis in patients with GBM, further investigation of additional signaling pathways and mechanisms that control GBM growth and invasion is needed to develop new therapies for significant improvement of clinical outcomes.

It has been well-established that protein glycosylation is one of the most important post-translational modifications [13]. Aberrations in protein glycosylation support tumor progression through various pathways [14]. Unique alterations in tumor-associated glycosylation may also serve as a distinct feature of cancer cells and therefore provide novel diagnostic and even therapeutic targets [15]. It has been reported that abnormal glycosylation significantly impacts the proliferation and invasion of gliomas [16]. O-mannosylation, one type of O-linked mannose glycosylation, has also been implicated in cancer and metastasis [17]. GDP-mannose is a key substrate for multiple glycosylation pathways, including O-mannosylation of alpha dystroglycan (α-DG), a component of the dystrophin-glycoprotein complex [18]. Recent works have focused on α-DG, a category of O-mannose modified protein, as its improper glycosylation is associated with cancer metastasis [19,20]. α-DG is widely expressed in many tissues, especially the brain and muscle; however, the necessity and sufficiency of O-mannosylation for glioma malignancy still remain to be determined [18].

GDP-mannose pyrophosphorylase B (GMPPB) is a cytoplasmic protein and, along with GMPPA, is one of the two key functional subunits for the enzyme GDP-mannose pyrophosphorylase (GDP-MP), which catalyzes the reaction of GTP and Man-1P to form GDP-mannose and diphosphate (PPi) [13]. Maintaining a proper level of GDP-mannose is essential for human development [21,22,23,24]. Previous studies have shown that GMPPB mutations correlate with several congenital diseases, including severe congenital muscular dystrophy (CMD) with abnormalities in the brain and eye [25]. It has been verified in zebrafish that disruption of the interactions between GMPPA and GMPPB results in abnormal brain development and muscle [13] abnormalities. Given GMPPBs involvement in the glycosylation process, further investigation into the role of GMPPB in gliomas may inspire novel strategies for the treatment and diagnosis of gliomas.

The Hippo pathway has been mapped out over the last several decades of study [26,27]. Two key proteins involved in transcriptional control of the Hippo pathway, Yes-associated protein (YAP) and transcriptional co-activator with PDZ-binding motif (TAZ), have been implicated in the progression of multiple human cancers [28,29], including GBM tumorigenesis and multi-drug resistance [30,31]. Matrix metalloproteinases (MMPs) are a group of enzymes that are shown to mediate carcinogenesis in both a physiological and pathological manner [32]. Matrix metallopeptidase 3 (MMP3) is one of the most studied MMP enzymes, and its overexpression has been associated with tumor growth and invasion in various types of tumors, including breast cancer, prostate cancer, pancreatic cancer, and gliomas [31,32,33,34,35]. Here, we examined if silencing GMPPB could inhibit the proliferation and invasion of GBM and if such activities are mediated by activating the phosphorylation of YAP at ser127 and inhibiting the downstream target gene, MMP3.

## 2. Results

### 2.1. GMPPB Is Upregulated in GBM Tumors and Correlated with Higher WHO Grades and Poor Prognosis

**Figure 1:**
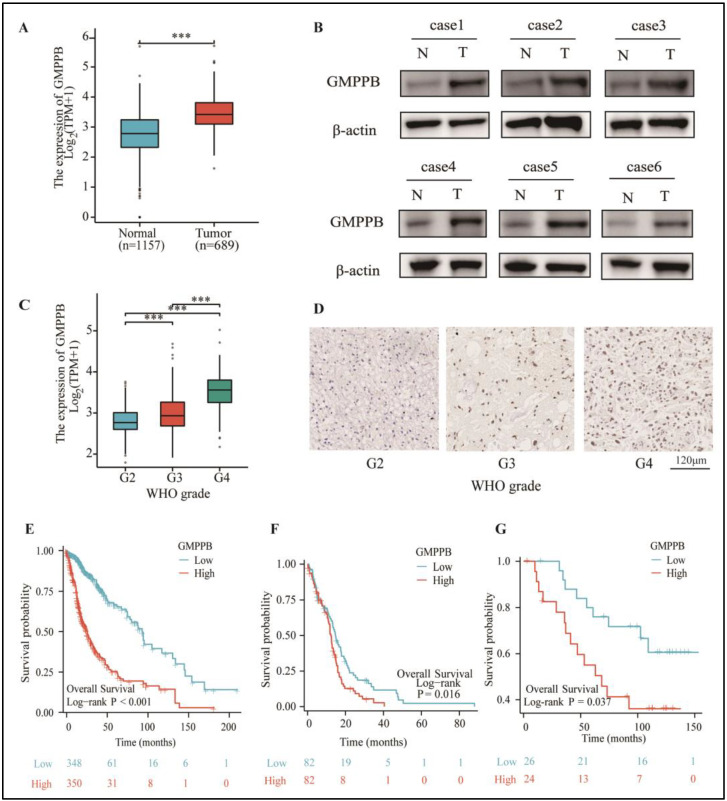
GMPPB is upregulated in GBM tumors and correlates with higher WHO grades and a poor prognosis. (**A**) The Cancer Genome Atlas (TCGA) dataset and Genotype-Tissue Expression (GTEx) dataset analysis of the GMPPB mRNA expression levels in glioma tumors and normal brain tissues *** *p* < 0.001, normal tissues: n = 1157; tumors: n = 689 (**B**) Immunoblot analysis of GMPPB in six glioma tumor samples and their adjacent normal brain tissues using β-actin was used as a loading control. (**C**) TCGA dataset analysis of the GMPPB mRNA expression levels in different glioma WHO grades: *** *p* < 0.001, G2: n = 224, G3: n = 245, G4: n = 168. (**D**) IHC staining of GMPPB in different WHO-grade glioma tumors. Scale bar = 120 μm. (**E**) TCGA dataset analysis of the relationship between the expression levels of GMPPB and the overall survival of glioma patients, *p* < 0.001, n = 698 (**F**) TCGA dataset analysis of the relationship between the expression levels of GMPPB and the overall survival of GBM patients, *p* = 0.016, n = 164 (**G**) Kaplan–Meier curve showing the relationship between the expression levels of GMPPB and the prognosis of glioma patients from the Department of Neurosurgery at Sun Yat-sen University Cancer Center, *p* = 0.037, n = 50.

Using the TCGA and GTEx databases to analyze GMPPB mRNA expression levels, we found that GMPPB was significantly upregulated in glioma tumors when compared to normal brain tissues (Figure 1A). We then examined the protein expression of GMPPB by Western blot in six patient samples, including 2 Grade II, 2 Grade III, and 2 Grade IV GBM tumors. Their adjacent “normal” brain tissue samples, resected to gain tumor exposure during surgery, were examined by our institutional neuropathologist. The small areas of normal tissue were included as references. The results showed that the glioma tumor samples expressed higher levels of GMPPB compared to their adjacent normal brain tissues (Figure 1B). To evaluate if GMPPB expression was related to the malignant grades of gliomas, we analyzed data from TCGA and found that there is a positive correlation between GMPPB expression and the WHO grades of gliomas and that GBM tumors expressed the highest levels of GMPPB (Figure 1C). We verified this finding by performing IHC staining on GBM tumor samples of different WHO grades. Our data confirmed that Grade IV GBM tumors had the highest expression of GMPPB as compared to Grade III and Grade II gliomas (Figure 1D). We also examined the correlation between the expression levels of GMPPB and overall survival. Analysis of mRNA expression in 698 cases in the TCGA database showed a reverse correlation between GMPPB expression and glioma (Figure 1E). We then extracted 164 GBM patients from the total of 698 glioma patients. The Kaplan–Meier curve results indicated that GMPPB correlates with a poor prognosis in GBM patients (Figure 1F). To support these results, we analyzed GMPPB expression in our 50 glioma clinical samples (collected from the Department of Neurosurgery at Sun Yat-sen University Cancer Center) (Table 1). As shown in Figure 1G, glioma patient samples with high GMPPB expression exhibited significantly poorer prognosis (*p* < 0.05) than those with low levels of expression. It is worthy of note that such differences were independent of the tumor grades.

### 2.2. Silencing GMPPB Inhibits GBM Cell Proliferation, Migration, and Invasion

**Figure 2:**
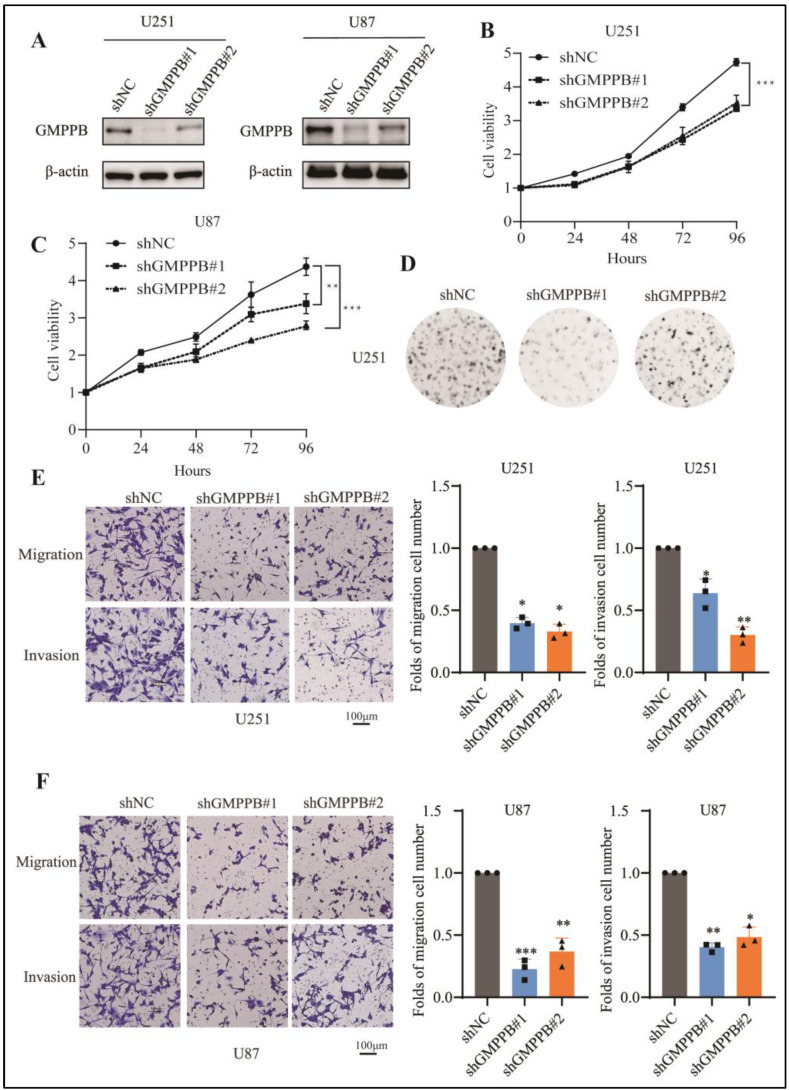
Silencing GMPPB inhibits GBM cell proliferation, migration, and invasion. (**A**) Immunoblot analysis of GMPPB in U251-shNC, U251-shGMPPB#1, U251-shGMPPB#2, U87-shNC, U87-shGMPPB#1, and U87-shGMPPB#2 cells using β-actin as a loading control (**B**) Effect of GMPPB knockdown on U251 GBM cell proliferation. n = 3. (**C**) Effect of GMPPB silencing on U87 GBM cell proliferation. n = 3. (**D**) Colony formation image of GMPPB knockdown on a U251 GBM cell (**E**) Representative images (**left**) and graphs (**right**) showing the effect of GMPPB silencing on U251 cell migration and invasion was assessed by transwell assays. n = 3. (**F**) Representative images (**left**) and graphs (**right**) showing the effect of GMPPB knockdown on U87 cell migration and invasion was assessed by transwell assays. n = 3. Folds of migration or invasion cell number = numbers of migrated or invaded cells in treatment groups/numbers of migrated or invaded cells in control groups. * *p* < 0.05; ** *p* < 0.01; *** *p* < 0.001. Scale bar = 100 μm.

To explore the biological role of GMPPB in GBM cells, we first performed loss-of-function studies by transducing U251 and U87 cells with two lentiviral shRNAs against GMPPB, i.e., shGMPPB#1 and shGMPPB#2. Lentiviral shRNA encoding a non-target (shNC) was included as a control. Western hybridization confirmed the near-complete inhibition of GMPPB expression by both shRNAs in the two lines (Figure 2A), accompanied by significantly inhibited cell proliferation and colony-forming efficiency (*p* < 0.05) in both U87 and U251 cells as assessed by the CCK-8 assay and the colony-forming assay, respectively (Figure 2B–D). Using a transwell assay, we also detected significantly reduced tumor migration and invasion following GMPPB knockdown (*p* < 0.05) (Figure 2E,F).

### 2.3. Overexpression of GMPPB Promotes GBM Cell Proliferation, Migration and Invasion

**Figure 3:**
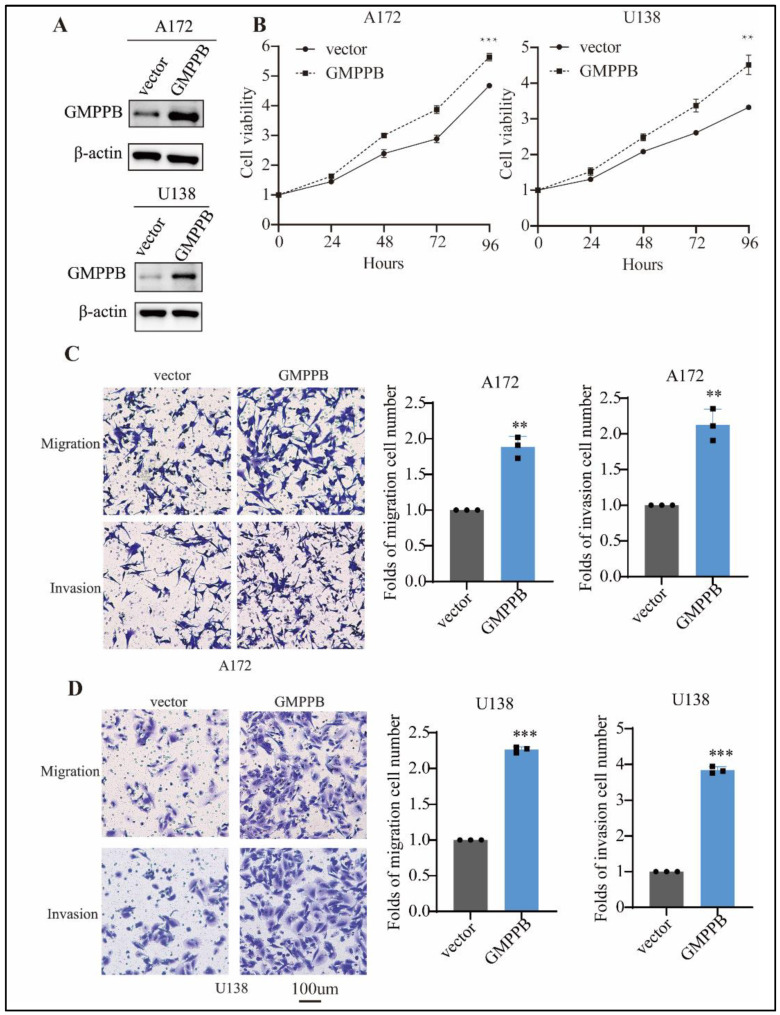
Overexpression of GMPPB promotes GBM cell proliferation, migration, and invasion. (**A**) Immunoblot analysis of GMPPB in A172-vector, A172-GMPPB, and U138-vector, U138-GMPPB cells using β-actin was used as a loading control. (**B**) Effect of GMPPB overexpression on A172 and U138 GBM cell proliferation. n = 3. (**C**) Representative images (**left**) and graphs (**right**) showing the effect of GMPPB overexpression on A172 cell migration as assessed by transwell assays. n = 3. (**D**) Representative images (**left**) and graphs (**right**) showing the effect of GMPPB overexpression on U138 cell migration and invasion as assessed by transwell assays. n = 3. Folds of migration or invasion cell number = numbers of migrated or invaded cells in treatment groups or numbers of migrated or invaded cells in control groups. ** *p* < 0.01; *** *p* < 0.001. Scale bar = 100 μm.

We next examined the effects of gain-of-function experiments on the A172 and U138 lines. These two lines were chosen because they endogenously expressed lower levels of GMPPB compared to U251 and U87 [36]. Transduction with lentiviral pLVX-GMPPB led to significantly elevated protein expression of GMPPB (Figure 3A), which was accompanied by significantly promoted cell proliferation (*p* < 0.05) as well as enhanced tumor cell migration (1.9 and 2.1 fold) and invasion (2.1 and 3.8 fold) in A172 and U138 cells, respectively (Figure 3B–D). Altogether, these data involving four different GBM cell lines support the role of GMPPB in modulating cell proliferation, migration, and invasion.

### 2.4. Downregulation of GMPPB Inhibits Glioblastoma Growth in Xenograft Models

**Figure 4:**
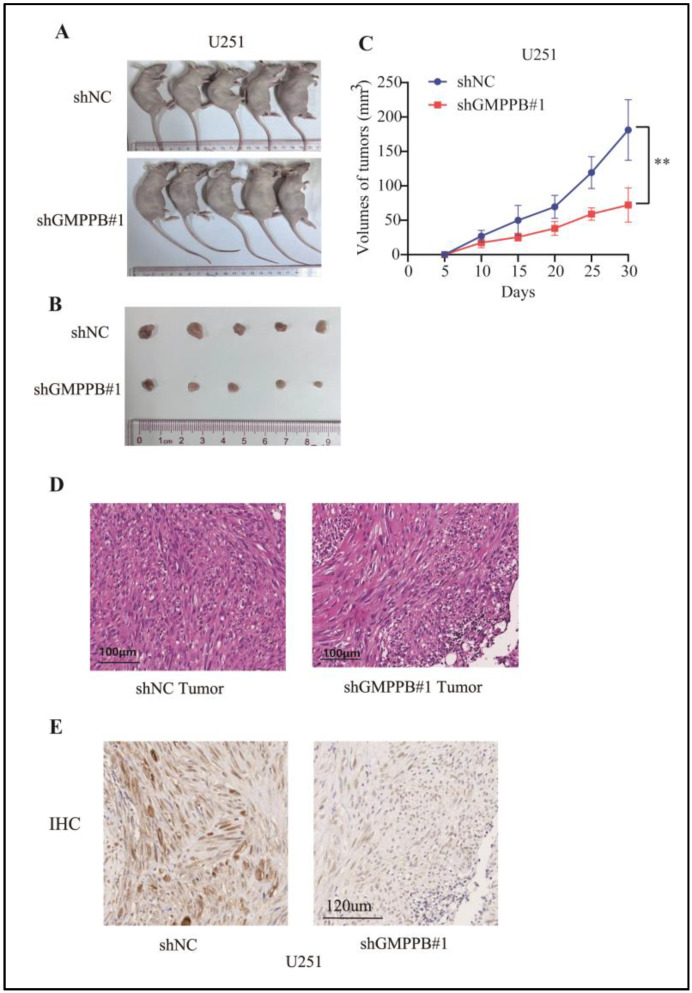
Downregulation of GMPPB inhibits glioblastoma growth in xenograft models. (**A**) Whole image of U251 negative control (shNC) group and GMPPB knockdown cells (shGMPPB#1) group xenograft mice after being euthanized (**B**) The two groups of tumors were isolated and compared. (**C**) The growth curves of xenograft tumors formed by U251 shNC or U251-shGMPPB#1 after their injection in nude mice The tumor volumes were measured every 5 days. Data are displayed as the mean ± SD (n = 5, ** *p* < 0.01). (**D**) HE staining was used to verify the formation and atypia of malignant tumors. Scale bar = 100 μm. (**E**) IHC was used to measure the protein levels of GMPPB in tumors from two groups. Scale bar = 120 μm.

We next examined whether downregulation of GMPPB led to similar effects in vivo as it had in vitro. U251 control cells and GMPPB knocked-down cells were inoculated subcutaneously into the right dorsal flanks of five mice each. Tumor growth was examined and measured every 5 days until day 30 post-inoculation. Compared with larger and more invasive tumors in the control groups, mice implanted with U251-shGMPPB#1 cells developed significantly smaller tumors with well-demarcated margins (*p* < 0.05) (Figure 4A–C). Histological examination revealed the growth of GBM with cellular atypia (H&E) and the loss of GMPPB expression as detected by IHC, thereby verifying the in vivo activities of silenced GMPPB (Figure 4D,E). Additionally, we have included HE staining images of the whole xenografts from 1 control and 1 shGMPPB group in Supplementary Figure S1.

### 2.5. Silencing GMPPB Inhibits the Proliferation and Invasion of Glioblastoma via Hippo/MMP3 Pathways

**Figure 5:**
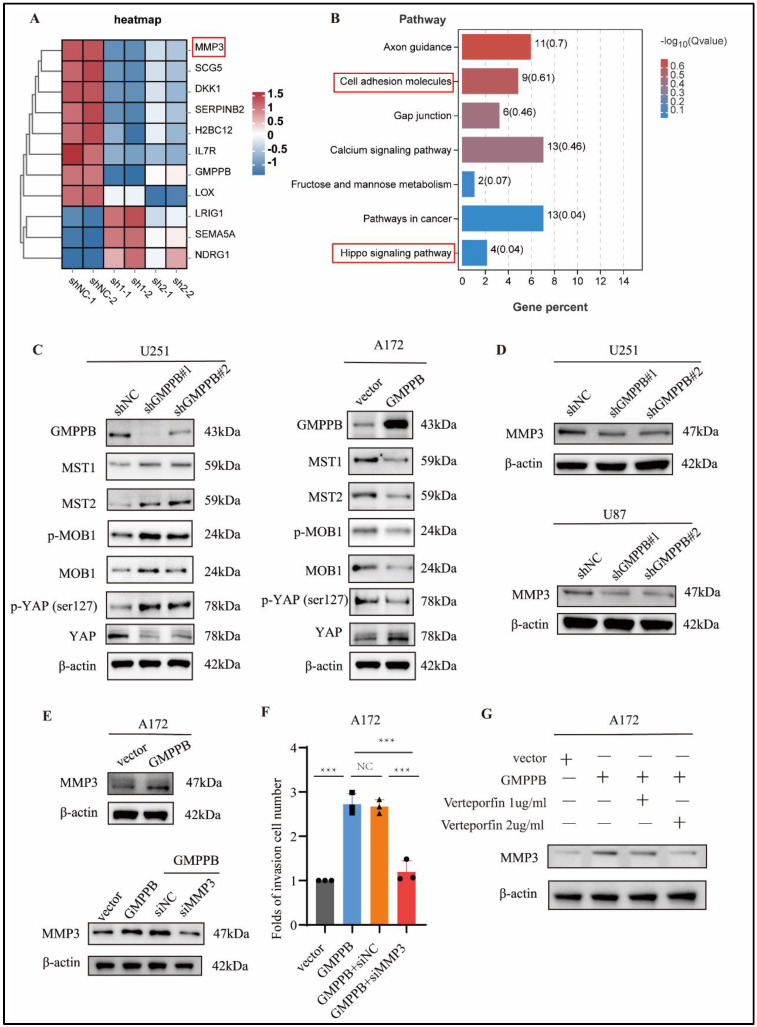
Silencing GMPPB inhibits the proliferation and invasion of glioblastoma cells via the Hippo/MMP3 pathway. (**A**) Representative heatmaps of transcriptome analysis indicate genes differentially regulated upon GMPPB knockdown in U87 cells, including MMP3 (*boxed in red*). (**B**) KEGG pathway analyses of GMPPB knockdown in U87 cells. Pathways potentially involved in invasion and migration, i.e., cell adhesion molecules, and pathways identified from the current study, i.e., Hippo signaling pathway, are highlighted in red boxes. (**C**) The indicated proteins in the Hippo pathway were analyzed by Western blotting in the GMPPB-knocked-down U251 cells and GMPPB-overexpressed A172 cells. (**D**) The protein MMP3 expression was displayed by Western blotting in the GMPPB-knocked-down U87, U251 cells, and GMPPB-overexpressed A172 cells. (**E**) MMP3 protein expression was displayed by Western blotting after si-NC and si-MMP3 RNA were transfected into A172 GMPPB-overexpressing cell lines. (**F**) Transwell assay analysis of the effect of si-NC or si-MMP3-transfected A172 GMPPB expression on GBM cell invasion *** *p* < 0.001. n = 3. Folds of invasion cell number = numbers of invaded cells in treatment groups/numbers of invaded cells in vector groups. (**G**) Verteporfin impacts the MMP3 protein expression displayed by Western blotting in GMPPB-overexpressing A172 cells. The concentration of verteporfin was 1 µg/mL and 2 µg/mL. β-actin was used as a loading control.

To explore the molecular mechanisms and signaling pathways essential for GMPPB-promoted GBM cell proliferation and invasion, we performed RNA-seq in GMPBB knockdown U87 cells and negative control U87 cells to identify the downstream targets of GMPPB (Figure 5A). The complete list of 548 differentially regulated genes from the RNA-seq was uploaded as Supplementary Table S1. Based on the results of RNA-seq, Kyoto Encyclopedia of Genes and Genomes (KEGG) pathway analysis revealed that gene sets that were significantly differentially regulated in GMPPB knockdown cells were mainly involved in cell adhesion, axon guidance, and Hippo signaling pathways (Figure 5B). Intriguingly, we also detected MMPs (Figure 5A,D), a gene that has been previously reported to play an important role in the malignancy of glioma cells [31,37,38,39], as one of the downstream target genes of GMPPB. Indeed, the expression of MMP3 was decreased by down-regulating GMPBB, while in GBM cells, over-expressing GMPPB increased the expression levels of MMP3 in GBM cells (Figure 5E). Activation of the Hippo pathways was another discovery. We found that in U251 cells, silencing GMPPB significantly increases the phosphorylation levels of both Mps one binder kinase activator 1 (MOB-1) and YAP (ser127), while the increased expression of GMPPB decreases the levels of p-MOB1 and p-YAP (ser127) in A172 cells (Figure 5C). Previous studies have revealed that phosphorylation of YAP at ser127 prevents YAP activation and translocation to the nucleus from the cytosol, resulting in inhibition of the expression of downstream target genes [40,41]. Additionally, several other important genes in the Hippo signaling pathways, such as Mammalian sterile-20-like 1/2 (MST1/2) and MOB1, were differentially expressed upon changes in GMPPB expression, and all of them contributed to the phosphorylation or dephosphorylation of YAP at the ser127 site (Figure 5C).

### 2.6. MMP3 Is Essential for GMPPB-Driven Cell Proliferation and Invasion and Is a Downstream Target Gene of the Hippo Pathway in GBM

We also detected MMP3 (Figure 5A,D) as one of the downstream target genes of GMPPB. Indeed, the expression of MMP3 was decreased by down-regulating GMPBB, while in GBM cells, over-expressing GMPPB increased the expression levels of MMP3 (Figure 5E). Given the important roles of MMP3 in the progression of glioma cells [31,37,38,39], we sought to functionally validate the role of MMP3 in mediating GMPPB-induced GBM proliferation and invasion and its relationship with the Hippo pathway. In order to answer this question, we knocked down MMP3 in our GMPPB-overexpressed A172 cells (Figure 5E) and found that downregulation of MMP3 reversed GMPPB-driven GBM cell proliferation (Figure S2) and invasion (Figure 5F). In order to further explore if MMP3 is one of the downstream target genes in the Hippo pathway, we applied the YAP inhibitor drug verteporfin (MCE, Concord, CA, USA, catalog number CL318952), which has been shown to disrupt YAP-TEAD interactions and to affect expression of its downstream target genes, such as Ki67, EGFR, CDH2, and ITGB1 [29,42]. We found that in A172 cell lines overexpressing GMPPB, verteporfin treatment inhibited the expression of MMP3 in a dose-dependent manner (Figure 5G). These results indicated that MMP3 is essential for GMPPB-driven cell proliferation and invasion. Since YAP is one of the key effector proteins (a transcriptional coactivator), our data suggested that MMP3 is a new downstream target gene of Hippo-YAP pathway in GBM [43,44].

## 3. Discussion

In this study, we investigate the biological role of GMPPB in GBM. We demonstrate that GMPPB is highly expressed in glioma tumors, particularly in GBM tumors. We also verify that high expression of GMPPB correlates with a poorer prognosis in patients with malignant gliomas. To further examine the molecular mechanisms that GMPPB plays in gliomas, we performed loss- and gain-of-function studies in four GBM cell lines. We found that silencing GMPPB inhibits the proliferation, migration, and invasion of GBM cell lines, and vice versa. Importantly, reduced expression of GMPPB inhibits GBM tumor growth in mice. Our research suggests GMPPB as a potential novel target for GBM treatment, which may be beneficial to patient survival. We further demonstrated that the Hippo/MMP3 axis plays an important role in GMPPB-promoted GBM malignancy.

Elucidating the signaling pathways regulated by GMPPB in GBM should provide novel insights about GBM biology. To identify pathways and downstream key target genes that GMPPB impacts most, we performed RNA sequencing after GMPPB was silenced in GBM cells. In addition to identifying Hippo pathways as importantly related to GMPPB, we further revealed that silencing GMPPB in GBM activates MST1/2 and increases phosphorylation of MOB1, which results in increased phosphorylation of YAP at the ser127 site. As reported previously, the phosphorylation of YAP at ser127 can prevent its translocation from the cytoplasm to the nucleus, leading to degradation and inactivation [45]. Conversely, we found that overexpression of GMPPB in GBM decreases the expression of MST1/2 and inhibits the phosphorylation of MOB1, causing the unphosphorylated YAP to escape from the cytoplasm and enter the nucleus, where it binds its transcriptional coactivator (TEAD). Our results build upon and extend previous findings that the interaction of YAP-TEAD prompts cancer cells to act as cancer stem cells, initiating DNA replication procedures, and triggering tumor proliferation, progression, and metastasis [28,30,40,45]. Despite the contributions provided by our study, the direct link between GMPPB and the Hippo pathway remains elusive, although there are multiple reports providing important clues. Previous studies have shown that overexpression or knockdown of wild-type GMPPB affects the glycosylation of α-DG and O-mannosylation [22,25]. Furthermore, impaired GMPPB function reduces the amount of GDP-mannose available for the O-mannosylation of α-DG [25]. Previous work has demonstrated that O-mannosylation shares some of the important biological functions of O-GlcNAcylation [46], which has been found to be involved in dysregulating the Hippo pathway in various cancer cell types [47,48]. Given the known functional similarities between O-GlcNAcylation and O-mannosylation, as well as what is known about the effects of O-GlcNAcylation on the Hippo pathway, we speculate that O-mannosylation may participate in GMPPB regulation of the Hippo pathway, which warrants further biological validation in GBM tumors.

In this study, we use subcutaneous xenograft models and established cell lines to verify the impacts of GMPPB knockdown on GBM growth. To further examine the impact of GMPPB and MMP3 knockdown in GBM tumors in a microenvironment similar to human brain tumors, we will expand our work to patient-derived orthotopic xenografts (PDOX) models as we have previously performed [49,50,51]. It is equally important to demonstrate that overexpression of GMPPB is strongly correlated with tumor cell proliferation, invasion, and metastasis in vivo. Since the parent A172 and U138 cell lines are known to be non-tumorigenic [52], future work will include GMPPB gain-of-function analysis in vivo using culture techniques that better replicate human GBM tumors, such as glioma stem or stem-like cells, together with the A172 and U138 cell lines. By replicating the human brain microenvironment, we are confident that the use of the PDOX model will provide critical details as to how GMPPB, Hippo, and MMP3 interact with each other to influence GBM progression. Worthy of note is that the overall levels of suppressed cell proliferation are <50% in our model system. Since the Hippo pathway has been implicated in multiple cell death modes, including apoptosis, autophagy, pyroptosis, and ferroptosis [53,54,55,56], it will be intriguing to explore the mechanisms of cell death, particularly in PDOX models, in the future. While the established cell lines do not exactly replicate the inter- and/or intra-tumoral heterogeneity as seen in cancer stem cell or stem-like GBM cell-derived cell cultures, PDOX models provide a useful resource to harvest and test glioma stem cells to support future efforts in validating GMPPB as a potential therapeutic target and testing new anti-cancer drugs against GMPPB in GBM [57]. Our study provides novel insights on the interplay progression of GMPPB regulation of the Hippo pathway to MMP3 through a series of functional validations. We show that reducing MMP3 expression can reverse the proliferation and invasion abilities of GMPPB-driven GBM. Most importantly, we demonstrate that MMP3 can be inhibited by the YAP/TAZ inhibitor verteporfin, which has entered clinical trials as a treatment for pancreatic cancer and glioma [58,59]. This finding is very exciting, as it demonstrates the power of biological studies in discovering new therapies.

## 4. Materials and Methods

### 4.1. Tumor Specimens and Cell Culture

Human surgical specimens were obtained from patients who underwent surgery at the Sun Yat-sen University Cancer Center (Guangzhou, China). This study was approved by the Institutional Ethical Review Board of Sun Yat-sen University Cancer Center (SL-B2023-039-01), and written informed consent was obtained from all patients. Final diagnoses of all the samples were made by our institutional neuropathologist (SR Liu) in accordance with the World Health Organization (WHO) criteria. The tumor tissues were snap frozen in liquid nitrogen and cryopreserved before use. A total of 50 glioma patient samples were included (Table 1).

Four human glioma cell lines (U87, U251, U138, and A172) were generously provided by Prof. Zhongping Chen (Sun Yat-sen University Cancer Center). They were cultured in DMEM (Corning, NY, USA) supplemented with 10% fetal bovine serum (FBS, Gibco, Grand Island, NY, USA). HEK293T embryonic kidney cells were obtained from the American Type Culture Collection (ATCC) and cultured in DMEM supplemented with 10% FBS. All cells were incubated at 37 °C and 5% CO_2_ and tested to rule out mycoplasma contamination before use.

### 4.2. Plasmid Construction

The human GMPPB (NM_013334.4) gene was cloned into a pLVX-puro vector following digestion with restriction endonucleases EcoRI-SmaI. The pLKO.1-puro vector was used to clone the shRNAs that target *GMPPB.* The sequences used for cloning the lentiviral shRNAs are CAGTGACGTGATCTGCGATTT for shGMPPB#1 and AGGGCTTCTGGATGGACATTG for shGMPPB#2.

### 4.3. Virus Production and Infection

Lentiviral vector pLVX-GMPPB or pLKO.1-shGMPPB and helper vectors psPAX2 and pMD.G-VSV-G were transfected into 293T cells by Lipofectamine 2000 reagent (Invitrogen, Carlsbad, CA, USA, catalog number 11668019) following the manufacturer’s instructions. The medium was changed with fresh DMEM/10% FBS after 24 h incubation. Then, the supernatant was collected after 24 h and filtered with a 0.45 μm nitrocellulose filter. The supernatant was used to infect glioma cells for 24 h and then selected with 2 μg/mL puromycin for 1 week. The stable pooled clones were verified by qRT-PCR and western blotting.

### 4.4. RNAi Treatment

siRNA transfection was performed according to the manufacturer’s instructions using Lipofectamine RNAi MAX transfection reagent (Invitrogen, Carlsbad, CA, USA) and 50 nM siRNA. The oligonucleotide target sequence for si-MMP3 is AGGATACAACAGGGACCAATT.

### 4.5. Quantitative RT-PCR

Total RNA was extracted using TRIzol reagent (Invitrogen) according to the manufacturer’s instructions and used as templates for reverse transcription into cDNA with the Reverse Transcription Kit (Takara, Tokyo, Japan). The levels of GMPPB (5′-3′ GGGAATCCGAATCTCCATGTC, 3′-5′ GTCTCAGAGAGTAGGTCACGG) and *MMP3* (5′-3′ CGGTTCCGCCTGTCTCAAG, 3′-5′ CGCCAAAAGTGCCTGTCTT) mRNA expression were determined by qRT-PCR using TB SYBR green Premix Ex Taq (Takara) and gene-specific primers, including two house-keeping genes, GAPDH (5′-3′ GGAGCGAGATCCCTCCAAAAT, 3′-5′ GGCTGTTGTCATACTTCTCATGG) and β-actin (5′-3′ CGCGAGAAGATGACCCAGAT, 3′-5′ GGGCATACCCCTCGTAGATG) were included as references.

### 4.6. Western Blotting

Cultured GBM cells were washed twice with ice-cold PBS and lysed by RIPA buffer (NCM Biotechnology, Suzhou, China) mixed with protease inhibitor cocktail (100×, NCM Biotechnology) and Phosphatase inhibitor (100×, NCM Biotechnology), followed by centrifugation at 12,000 rpm for 20 min at 4 °C to remove cell fragments. Twenty micrograms of protein were loaded and separated on a 10% sodium dodecyl sulfate–polyacrylamide gradient gel. The gels were transferred to Immobilon-P PVDF membranes (Millipore, Burlington, MA, USA), which were then blocked (1 h) in TBST (20 mM Tris-HCl, pH 7.4; 150 mM NaCl and 0.2% Tween-20) with 5% BSA (Sigma-Aldrich, Saint Louis, MO, USA) and incubated with diluted primary antibodies overnight at 4 °C. After using the secondary HRP-conjugated antibodies, the clarity ECL substrate (Biosharp, Hefei, China) was used for detection by a MiniChmei Chemiluminescence imager (SAGECREATION, Beijing, China).

Primary antibodies used were GMPPB Polyclonal Antibody (1:1000) (catalog number 15094-1, Proteintech, Rosemont, IL, USA), MMP3 Antibody (1:2000) (catalog number ab52915, Abcam, Cambridge, MA, USA), Hippo Signaling Antibody Sampler kit (1:1000) (catalog number 3579, Cell Signaling Technology, Danvers, MA, USA), and b-actin Antibody (1:10,000) (Cell Signaling Technology, catalog number 8457).

### 4.7. Cell Proliferation

Cell viability was assessed by the Cell Counting kit-8 (CCK-8; APExBIO, USA) assay and the colony formation assay, as we described previously. Briefly, the cells were plated into 96-well plates at a density of 2 × 10^3^ cells/100 μL/well, and the test was started 24 h later and lasted for 4 consecutive days. The medium in the well was discarded, and CCK-8 reagent was mixed with serum-free medium in advance and added into 96-well plates with 100 μL per well (CCK8 reagent: Serum-free medium = 10 μL: 90 μL per well). After incubation at 37 °C for 1.5 h, a 450 nm OD value was detected by spectrophotometric measurements. For the colony formation assay, cells were collected and plated in six-well plates in an incubator for 10 days. Next, cells were fixed with 4% paraformaldehyde for 15 min and stained with crystal violet for 15 min. The images of colonies in each well were collected.

### 4.8. Migration and Invasion

The evaluation of migration and invasion of GBM cells was performed using Boyden chambers containing 24-well Transwell plates (BD Inc., San Jose, CA, USA) with a pore size of 8 μm. All experiments were performed in duplicate and repeated three times. For the migration assay, the transwell upper chambers (8 μm pore size) were seeded with 1 × 10^5^ (U87, U251) cells or 8 × 10^4^ (A172, U138) cells in 100 μL of serum-free DMEM without an extracellular matrix coating. DMEM containing 10% FBS was added to the lower chamber. After 18 h of incubation, the cells on the bottom surface of the 8 µm filter were fixed, stained, and examined using a microscope. For the invasion assay, the transwell upper chamber (8 μm pore size) was coated with 50 μL of 1:8 diluted Matrigel (Corning, NY, USA) at 37 °C for 2 h to obtain Matrigel solidified. Then, 1 × 10^5^ (U87, U251) cells or 8 × 10^4^ (A172, U138) cells in 100 μL of serum-free DMEM were added to the upper chamber, and the lower chamber was filled with culture medium containing 20% FBS. After 24 h of incubation at 37 °C in 5% CO_2_, the cells were fixed, stained, and observed as described for the migration assays.

### 4.9. Animal Experiments

All animal procedures were performed following the “Guide for the Care and Use of Laboratory Animals” and the “Principles for the Utilization and Care of Vertebrate Animals” and a protocol approved by the Animal Research Committee of Sun Yat-sen University Cancer Center (L102042022080K). Four-week-old female BALB/c nude mice were purchased from Jiangsu GemPharmatech Lab Animal Technology Co., LTD. For subcutaneous xenograft models, 1 × 10^6^ control or GMPPB knockdown U251 cells were suspended in 100 µL of PBS and implanted into the flanks of nude mice. Tumor sizes were measured every 5 days, and tumor volumes were calculated with the formula Volume = (length × width^2^)/2. After 30 days, the animals were sacrificed, and their subcutaneous tumors were removed and imaged. All the dissected tumor tissue samples were paraffin-embedded, sectioned, and stained for histopathological analysis.

### 4.10. Immunohistochemistry Staining (IHC) and H Score

IHC staining was performed on 3 μm sections. The glioma tissue sections were heated at 65 °C for dewaxing, followed by antigen retrieval in citrate antigen retrieval solution. After blocking with goat serum at 37 °C for 30 min, the primary anti-GMPPB antibody (LSBio, Seattle, WA, USA, LS-C81311) was diluted in 1:200 and then incubated at 4 °C overnight in a humidified container. After three washes with TBS, the tissue slides were treated with a Dako real-imaging peroxidase detection system according to the manufacturer’s instructions (Dako, Glostrup, Denmark).

The IHC images were acquired by using a digital pathology slide scanner (KFBIO, KF-PRO-020). Then, the H score of the images was evaluated by the HALO image analysis system (Indica Labs, Albuquerque, NM, USA). To be more detailed, after the images were obtained by the HALO system, most of the cells in the slides would be collected and categorized into different levels. For example, negative, weak, moderate, and strong are all based on the staining degree. Then, the analysis system will record the cell proportion of each staining degree and eventually use it for Histochemistry score (H-score) calculation.

### 4.11. Bioinformatics Analysis

To examine the expression of GPPB in a large cohort of patient tumors, we extracted data from the TCGA (https://tcga-data.nci.nih.gov, accessed on 10 February 2023) and GTEx (https://gtexportal.org, accessed on 10 February 2023) databases and applied R software (version 4.2.1) to complete the Bioinformatics analysis. Additionally, we retrieved GMPPB TPM RNA-seq data of glioma and normal tissues from UCSC XENA (https://xenabrowser.net/datapages/ accessed on 10 February 2023) unified by the Toil process [60], applied the statistics package, and used the car package for statistical analysis. Before we applied the ggplot2 package for data visualization, updated WHO grading of the tumors was obtained from the supplementary data described by Ceccarelli et al. [61]. To examine the impact of GMPPB expression on patient survival, we used the TCGA database to download TCGA GBM and TCGA LGG project STAR processes of RNAseq data and extract the TPM format of the data, which was then correlated with the prognosis reported by Liu et al. [62] The survival package was applied to test the proportional risk hypothesis and fit survival regression.

### 4.12. Statistical Analysis

The significance of differences between groups was analyzed by the Student’s *t* test and among multiple groups with analysis of variance (ANOVA) using SPSS (Version 16.0, IBM, Armonk, NY, USA) and GraphPad Prism (Version 9.0, La Jolla, CA, USA). The correlations between GMPPB expression and overall survival curves were assessed using Kaplan-Meier analysis. *p* values < 0.05 were considered statistically significant.

## 5. Conclusions

In this study, we demonstrate that GMPPB is important in GBM growth and metastasis and identify it as a novel prognosis biomarker and a potential therapeutic target in glioma. Additionally, we characterized the Hippo/MMP3 axis as an important pathway during GMPPB-driven GBM progression and further showed that inhibiting MMP3 could reverse GMPPB-driven invasion. We also demonstrated that pharmacological inhibition of YAP/TAZ with Verteporfin can inhibit MMP3 expression in GBM.

## Figures and Tables

**Table 1:** Summarize the 50 glioma patients involved in the GMPPB expression and prognosis analysis.

		Male	Female	Total
**WHO Grade**	Grade I	4	1	5
	Grade II	7	12	19
	Grade III	15	2	17
	Grade IV	2	5	7
	NA			2
**Age**	Age < 40	14	18	32
	Age > 40	7	11	18
**GMPPB** (Median expression as cut-off value)	Low expression	14	12	26
	High expression	15	9	24

## Data Availability

The datasets used and/or analyzed during the current study are available from the corresponding author on reasonable request.

## Supplementary Materials

The following supporting information can be downloaded at: https://www.mdpi.com/article/10.3390/ijms241914707/s1.

## Abbreviations

GMPPB, GDP-mannose pyrophosphorylase B; GDP-MP, GDP-mannose pyrophosphorylase; GBM, Glioblastoma multiforme; YAP, Yes-associated protein; MMP3, Matrix metallopeptidase 3.
