# Supplementary figures and images for: Silencing GMPPB Inhibits the Proliferation and Invasion of GBM via Hippo/MMP3 Pathways
Source: Int J Mol Sci. 2023 Sep 28;24(19):14707. doi: 10.3390/ijms241914707 (PMC10572784; doi:10.3390/ijms241914707)

shGMPPB#1

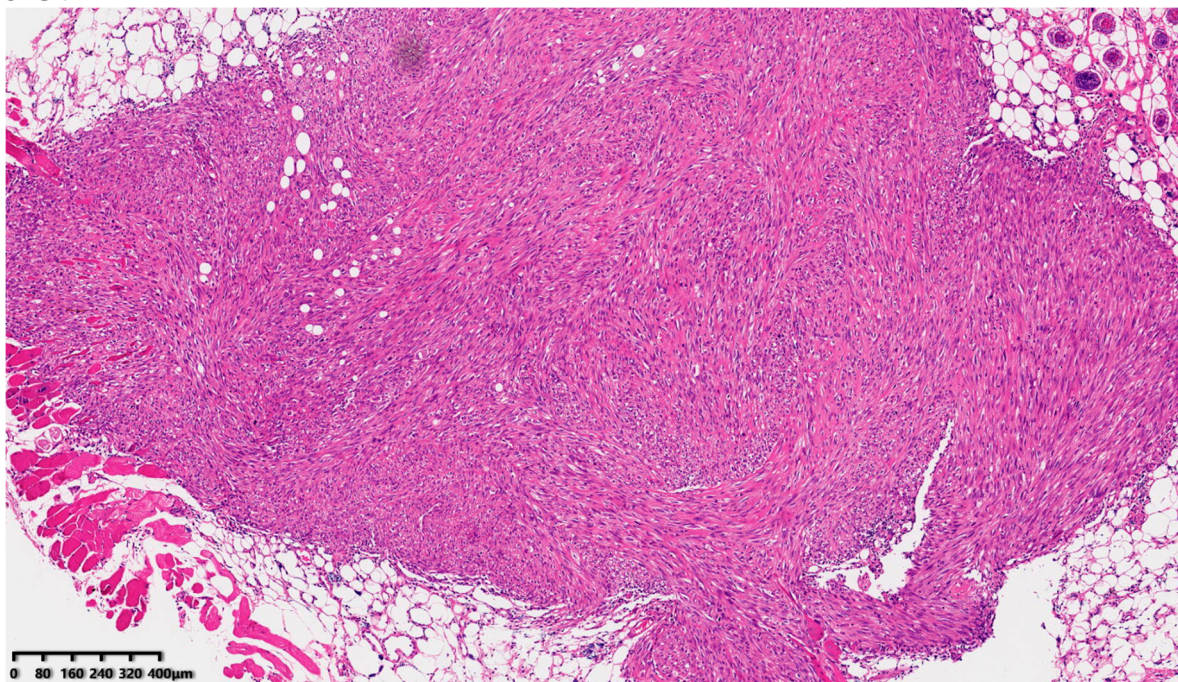

shNC

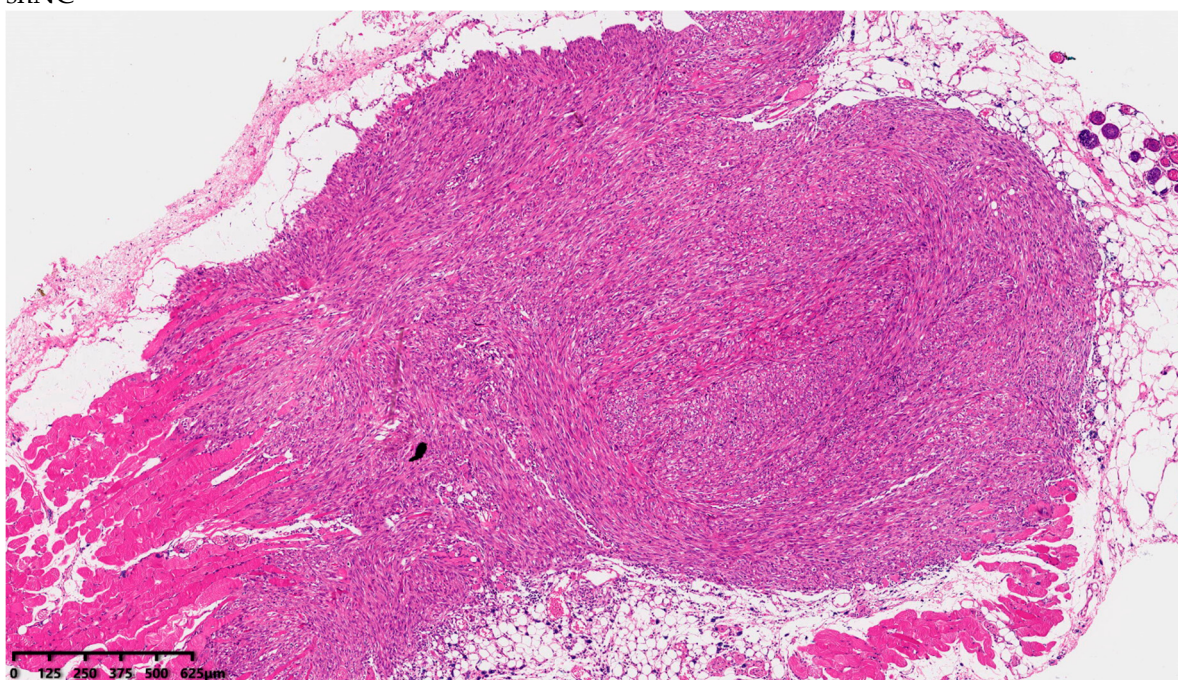

Supplement: Supplementary file 1 [file ijms-24-14707-s001.zip › Supplementary Figure S1.pdf]

A172

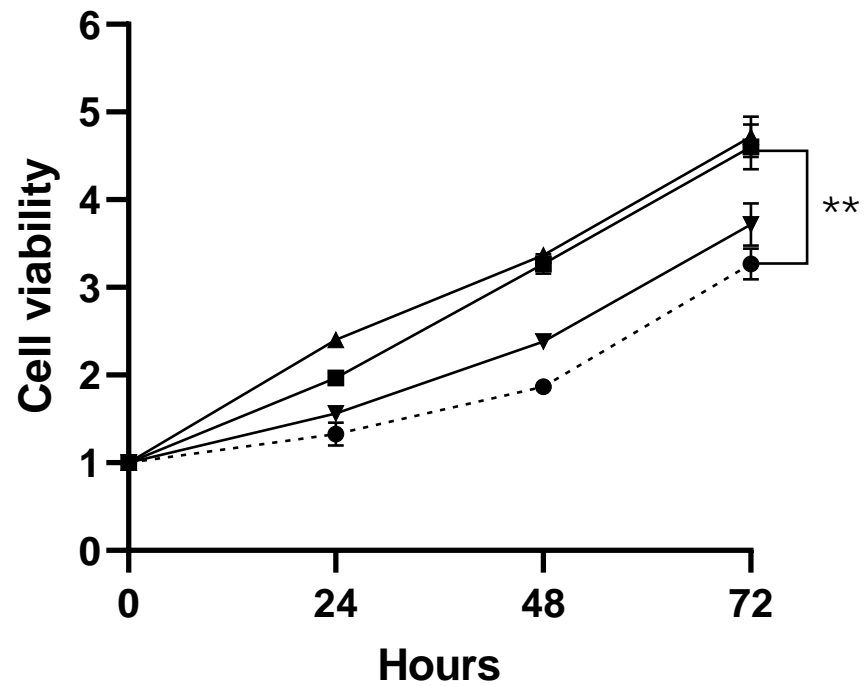

- ▲— GMPPB
- GMPPB+siNC
- ▼— vector
- GMPPB+siMMP3

Supplement: Supplementary file 1 [file ijms-24-14707-s001.zip › Supplementary Figure S2.pdf]
